# Fashioning esophagogastric anastomosis in robotic Ivor-Lewis esophagectomy: a multicenter experience

**DOI:** 10.1007/s00423-024-03290-3

**Published:** 2024-03-22

**Authors:** Marco Milone, Paolo Pietro Bianchi, Fabio Cianchi, Andrea Coratti, Anna D’Amore, Giovanni De Manzoni, Carlo Alberto De Pasqual, Giampaolo Formisano, Elio Jovine, Luca Morelli, Mariafortuna Offi, Andrea Peri, Andrea Pietrabissa, Fabio Staderini, Angela Tribuzi, Simone Giacopuzzi

**Affiliations:** 1 Department of Clinical Medicine and Surgery, ″Federico II″ University of Naples, Via Sergio Pansini, 5, 80131 Naples, Italy; 2 Department of Health Science, University of Milan, 20142 Milan, Italy; 3 Chirurgia Dell’Apparato Digerente Azienda Ospedaliero-Universitaria Careggi, Florence, Italy; 4 Misericordia Hospital of Grosseto, Grosseto, Italy; 5 General and Upper GI Surgery Division, Department of Surgery, University of Verona, Verona, Italy; 6 Department of General Surgery, IRCCS, Azienda Ospedaliero-Universitaria Di Bologna, Maggiore Hospital, 40133 Bologna, Italy; 7 General Surgery Unit, Department of Translational Research and New Technologies in Medicine and Surgery, University of Pisa, Pisa, Italy; 8 Fondazione IRCCS Policlinico San Matteo, Pavia, Italy; 9 Department of Surgery, University of Pavia, Pavia, Italy

**Keywords:** Esophagogastric, Anastomosis, Robotic, Ivor-Lewis, Esophagectomy

## Abstract

**Background:**

The aim of the present study is to compare outcomes of the robotic hand-sewn, linear- and circular-stapled techniques performed to create an intrathoracic esophagogastric anastomosis in patients who underwent Ivor-Lewis esophagectomy.

**Methods:**

Patients who underwent a planned Ivor-Lewis esophagectomy were retrospectively analysed from prospectively maintained databases. Only patients who underwent a robotic thoracic approach with the creation of an intrathoracic esophagogastric anastomosis were included in the study. Patients were divided into three groups: hand-sewn-, circular stapled-, and linear-stapled anastomosis group. Demographic information and surgery-related data were extracted. The primary outcome was the rate of anastomotic leakages (AL) in the three groups. Moreover, the rate of grade A, B and C anastomotic leakage were evaluated. In addition, patients of each group were divided in subgroups according to the characteristics of anastomotic fashioning technique.

**Results:**

Two hundred and thirty patients were enrolled in the study. No significant differences were found between the three groups about AL rate (*p* = 0.137). Considering the management of the AL for each of the three groups, no significant differences were found. Evaluating the correlation between AL rate and the characteristics of anastomotic fashioning technique, no significant differences were found.

**Conclusions:**

No standardized anastomotic fashioning technique has yet been generally accepted. This study could be considered a call to perform ad hoc high-quality studies involving high-volume centers for upper gastrointestinal surgery to evaluate what is the most advantageous anastomotic technique.

## Introduction

Esophageal cancer is the seventh most common cause of cancer morbidity and the sixth leading cause of cancer-related death [1–3]. The milestone for the treatment of esophageal cancer is radical esophagectomy with lymphadenectomy [4]. Totally minimally invasive (laparoscopy and thoracoscopy) Ivor–Lewis (TMIIL) has increased in popularity, providing the well-known advantages on recovery of the minimally invasive procedures. Furthermore, several experiences [5–8] have reported safety and efficacy of minimally invasive esophagectomy, showing similar oncologic results and long-term recurrence rate [9–11].

In the new era of minimally invasive surgery, a robotic approach could offer the chance to overcome technical difficulties of laparoscopic technique due to its three-dimensional view and its EndoWrist® technology. Although robotic surgery could be considered the gold standard only for the treatment of prostate cancer, it has gained popularity in many fields of surgical practice arousing the interest of several surgeons [12–18]. Regarding TMIIL, the creation of an intrathoracic esophagogastric anastomosis is one of the most critical phases and robotic approach seems to facilitate the surgeon especially in this step. On the other hand, no standardized technique has yet been generally accepted. Various approaches have been used and they are basically divided into hand-sewn, mechanical and semi-mechanical, the last two of which involve the use of a stapler, either linear or circular. Circular- (CS) and linear-stapled (LS) are the two most commonly used anastomotic techniques [19]. In LS, the hand-suturing of the anterior aspect of the anastomosis is the most technically difficult phase while in CS this role falls to the complexity of performing the purse-string suture fixing the anvil.

The aim of the present study is to compare outcomes of the robotic hand-sewn-, linear- and circular-stapled techniques and to help robotic surgeons in choosing the best intrathoracic esophagogastric anastomosis technique.

## Materials and methods

Patients with middle and lower esophageal cancers or Siewert type 1 or 2 esophagogastric junction carcinoma who underwent a planned Ivor-Lewis esophagectomy at seven high-volume Italian centers for upper gastrointestinal surgery were retrospectively analysed from prospectively maintained databases. Only patients who underwent a robotic thoracic approach with the creation of an intrathoracic esophagogastric anastomosis were included in the study to compare outcomes of the robotic hand-sewn-, linear- and circular-stapled techniques.

A written informed consent was obtained from each patient enrolled in the analysis. The study protocol conforms to the ethical guidelines of the 1975 Declaration of Helsinki.

All operations were performed by senior surgeons experienced in minimally invasive esophagectomy using different techniques to perform the intrathoracic esophagogastric anastomosis (hand-sewn-, circular- or linear-stapled). To minimize the bias related to the presence of different surgeons, only procedures performed by experts of high-volume Italian centers for upper gastrointestinal surgery were considered. In addition, only robotic interventions were included to specifically evaluate the best anastomotic technique in the robotic setting.

Demographic information and surgery-related data were extracted. Demographic information included sex, age, BMI, ASA score and comorbidities (hypertension, diabetes, congestive heart failure and chronic obstructive pulmonary disease COPD). Surgery-related data involved AL rate.

### Outcomes

Patients were divided in three groups according to the anastomotic technique used (hand-sewn-, circular- and linear-stapled).

The primary outcome was the rate of AL in the three groups. The diagnosis of AL was based on CT scan and/or upper GI endoscopy which were performed in case of leak suspicion, based on clinical symptoms, routine laboratory tests and/or chest X-ray. Moreover, the rate of grades A, B and C anastomotic leakage in the three groups were evaluated. AL was defined as grade A when it was treated conservatively with no change in patients’ management, it was defined as grade B when it was treated with invasive intervention such as endoscopic or radiological intervention other than repeat surgical intervention and it was defined as grade C when it was treated with surgical intervention.

In addition, patients of each group were divided in subgroups according to the characteristics of anastomotic fashioning technique. In details, patients of the hand-sewn anastomosis group were divided in two subgroups according to the type of suture thread used (barbed, non-braided); patients of the circular-stapled anastomosis group were divided in four subgroups according to the circular-stapled technique (manual, purse-string device) and to the stapler diameter (25 mm, 29 mm); patients of the linear-stapled anastomosis group were divided in nine subgroups according to the stapler type (mechanical, electric), to the stapler length (45 mm, 60 mm), to the layer of defect closing (single, double) and to the type of suture thread used for defect closing (barbed, braided, non-braided).

During postoperative course, patients were evaluated with clinical monitoring and daily blood tests. After the discharge, the patients were submitted to a check after 7, 30, 60 and 90 days.

### Surgical technique

All procedures included in the study were performed according to the principles of a radical esophagectomy with the lymphadenectomy [4].

Only patients who underwent a robotic thoracic approach with the creation of an intrathoracic esophagogastric anastomosis were included in the study.

### Anastomotic technique

No standardized technique has yet been generally accepted to perform the esophagogastric anastomosis. The approaches used involve hand-sewn-, circular- (CS) and linear-stapled (LS).

The esophagogastric anastomosis is performed above the level of the azygos arch.

**Fig. 1:**
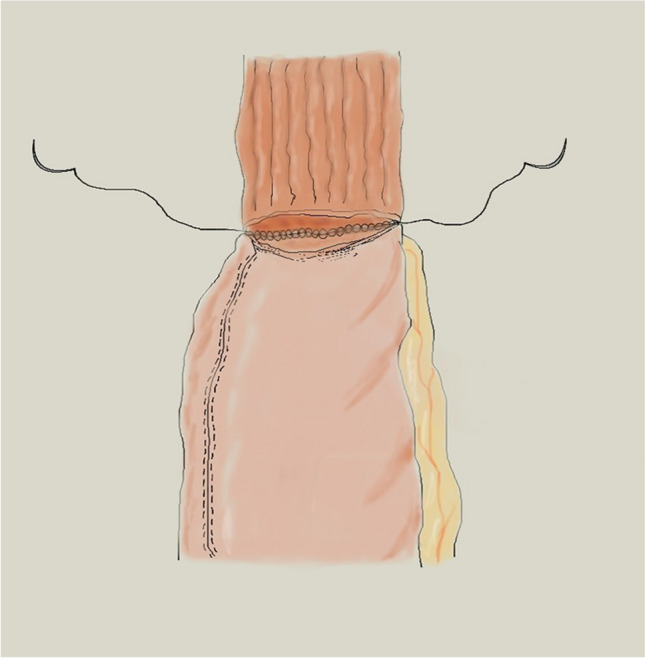
Robotic hand-sewn anastomotic technique

In detail, during the robot-sewn esophagogastric anastomotic fashioning technique, once the posterior aspect of the anastomosis is complete, the nasogastric tube is placed under direct vision inside the stomach distally to the anastomosis to accomplish the closure of anterior surface. Particularly, a double-layer closure using a running barbed suture in the first layer for posterior surface and a single layer using a running barbed suture for anterior surface of the anastomosis are performed (Fig. 1).

**Fig. 2:**
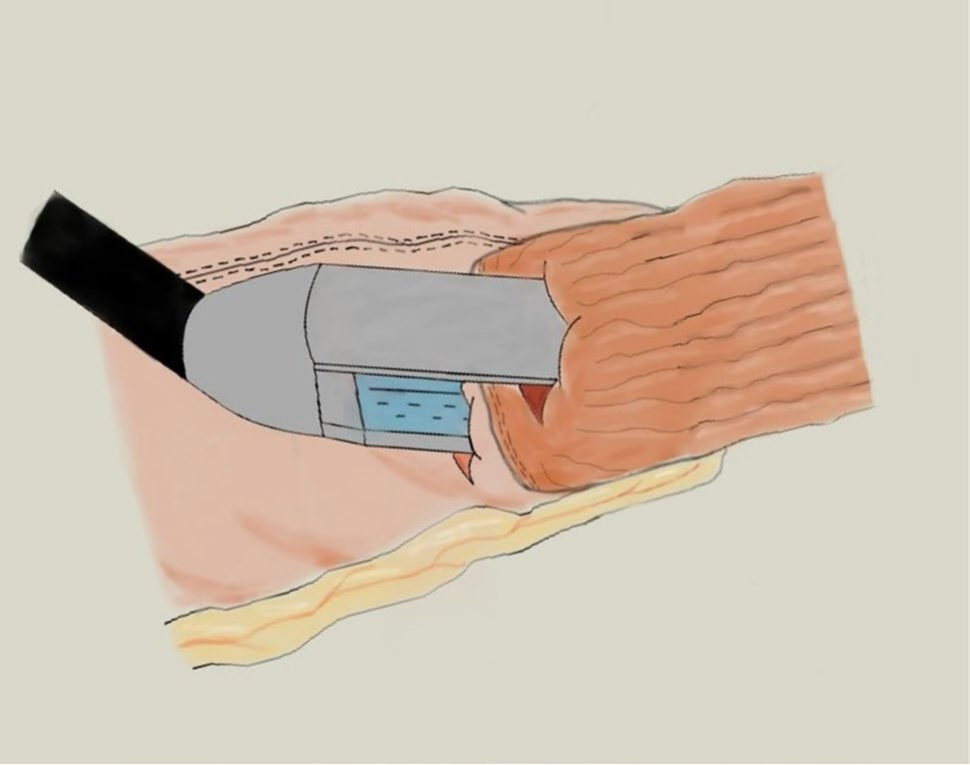
Linear-stapling technique

The esophagogastric side-to-side anastomosis is performed using a linear stapler and the enterotomies are closed by hand-sewn sutures, after passing the nasogastric tube in the conduit under direct vision (Fig. 2).

**Fig. 3:**
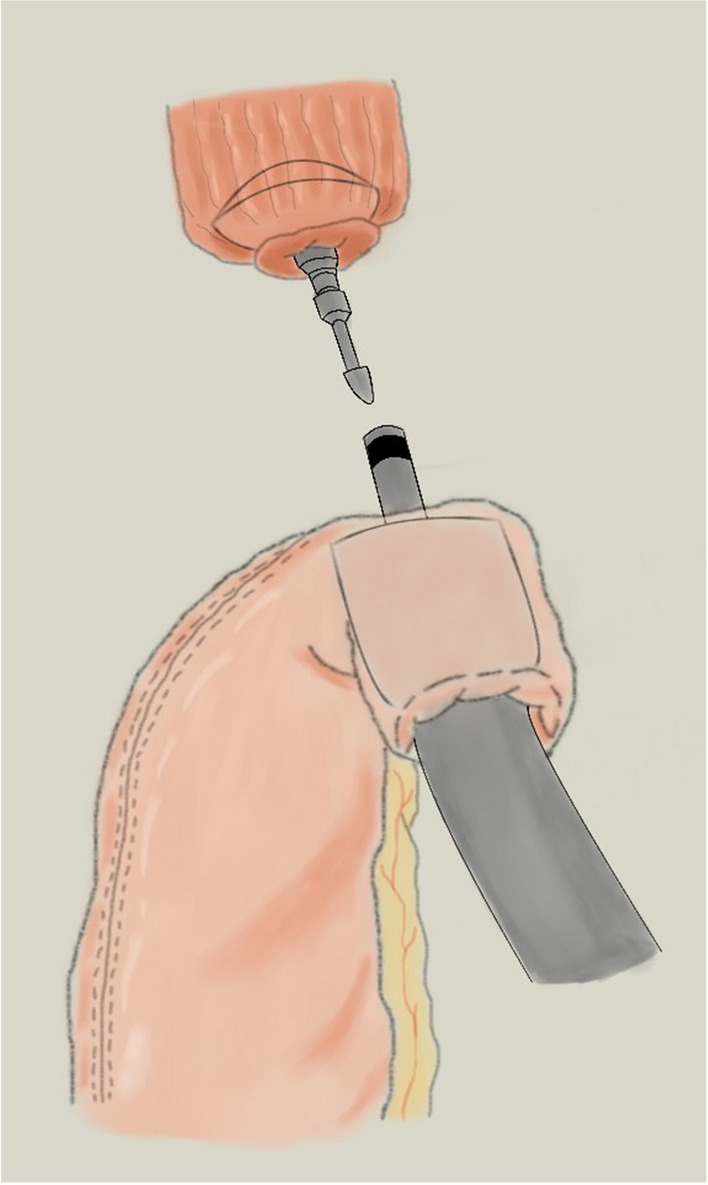
Circular-stapling technique

The esophagogastric end-to-side anastomosis is performed using a circular stapler. In details after esophagus transection, the anvil is secured and the circular stapler is introduced into the gastric tube with the tip of the stapler emerging at the mesenteric side of the gastric conduit, after which the two stapler parts are aligned and the stapler is fred. A linear stapler is then used to close the open end of the gastric tube, and the anastomosis is oversewn with two sutures (Fig. 3).

A leak test is performed with methylene blue.

### Statistical analysis

Statistical analysis was performed using the SPSS 28 system (SPSS Inc., Chicago, IL, USA). Continuous data were expressed as mean ± standard deviation, and categorical variables were expressed as percentages. The ANOVA test was performed to compare continuous variables. The chi-square test was employed to analyse categorical data.

## Results

Two hundred and ninety-three patients with middle and lower esophageal cancer or Siewert type 1 or 2 esophagogastric junction carcinoma, who underwent a planned Ivor-Lewis esophagectomy at seven high-volume Italian centers for upper gastrointestinal surgery, were eligible for study inclusion but 63 were excluded because two patients underwent an open technique and 61 did not receive an intrathoracic robotic esophagogastric anastomosis. Thus, 230 patients were enrolled in the analysis.

In details, patients were divided into three groups based on the technique used to perform the esophagogastric anastomosis; thus, the hand-sewn anastomosis group involved 64 patients (27.8%), the circular-stapled anastomosis group involved 71 patients (30.9%) and the linear-stapled anastomosis group involved 95 patients (41.3%).

Demographic and tumor characteristics of the three compared groups are showed in Table 1.

**Table 1:** Demographic characteristics and tumor characteristics of the three compared groups

	Hand-sewn (*n* = 64; 27.8%)	Circular-stapler (*n* = 71; 30.9%)	Liner-stapler (*n* = 95; 41.3%)	*P*
Characteristics				
M/F (%)	54/10 (84.4/15.6)	68/3 (95.8/4.2)	83/12 (87.4/12.6)	0.081
Age	66.4 ± 10.6	63.6 ± 9.6	65.6 ± 9.1	0.210
BMI	25.5 ± 4.1	27.1 ± 3.9	26.7 ± 3.9	0.065
ASA 1 (%) 2 (%) 3 (%) 4 (%)	15 (23.4%) 16 (25%) 14 (21.9%) 19 (29.7%)	13 (18.3%) 24 (33.8%) 18 (25.4%) 16 (22.5%)	22 (23.2%) 30 (31.6%) 25 (26.3%) 18 (18.9%)	0.714
Hypertension (%)	41 (64.1%)	33 (46.5%)	46 (48.4%)	0.079
Diabetes (%)	11 (17.2%)	12 (16.9%)	16 (16.8%)	0.998
Congestive heart failure (%)	7 (10.9%)	9 (12.7%)	8 (8.4%)	0.667
COPD (%)	8 (12.5%)	10 (14.1%)	18 (18.9%)	0.508
Smoking (%)	25 (39.1%)	18 (25.3%)	21 (22.1%)	0.227
T Tis (%) T0 (%) T1 (%) T2 (%) T3 (%) T4 (%)	0 6 (9.4%) 5 (7.8%) 16 (25%) 37 (57.8%) 0	1 (1.4%) 16 (22.5%) 6 (8.5%) 17 (24%) 30 (42.2%) 1 (1.4%)	2 (2.1%) 13 (13.7%) 12 (12.6%) 19 (20%) 46 (48.4%) 3 (3.2%)	0.254
N N0 (%) N1 (%) N2 (%) N3 (%)	29 (45.3%) 14 (21.9%) 8 (12.5%) 13 (20.3%)	40 (56.3%) 15 (21.1%) 12 (16.9%) 4 (5.6%)	53 (55.8%) 24 (25.3%) 8 (8.4%) 10 (10.5)	0.119
M Mx (%) M0 (%) M1 (%)	2 (3.1%) 62 (96.9%) 0	0 70 (98.6%) 1 (1.4%)	4 (4.2%) 91 (95.8%) 0	0.275
Neoadjuvant therapy (%)	28 (43.7%)	38 (53.5%)	42 (44.2)	0.411

Categorical variables are expressed as numbers and (percentages), while continuous variables are expressed as mean ± SD

*M* male, *F* female, *BMI* body mass index, *ASA score* American Society of Anesthesiology score, *COPD* chronic obstructive pulmonary disease

The comparative analysis between the three groups showed no differences about demographic data including sex, age, BMI, ASA score, comorbidities (hypertension, diabetes, congestive heart failure and chronic obstructive pulmonary disease COPD), smoking habit and about pTNM (Table 1). Moreover, 108 out of 230 patients underwent neoadjuvant therapy, of which 28 patients (43.7%) belonged to the hand-sewn anastomosis group, 38 (53.5%) to the circular-stapled group and 42 (44.2%) to the linear-stapled anastomosis group.

**Fig. 4:**
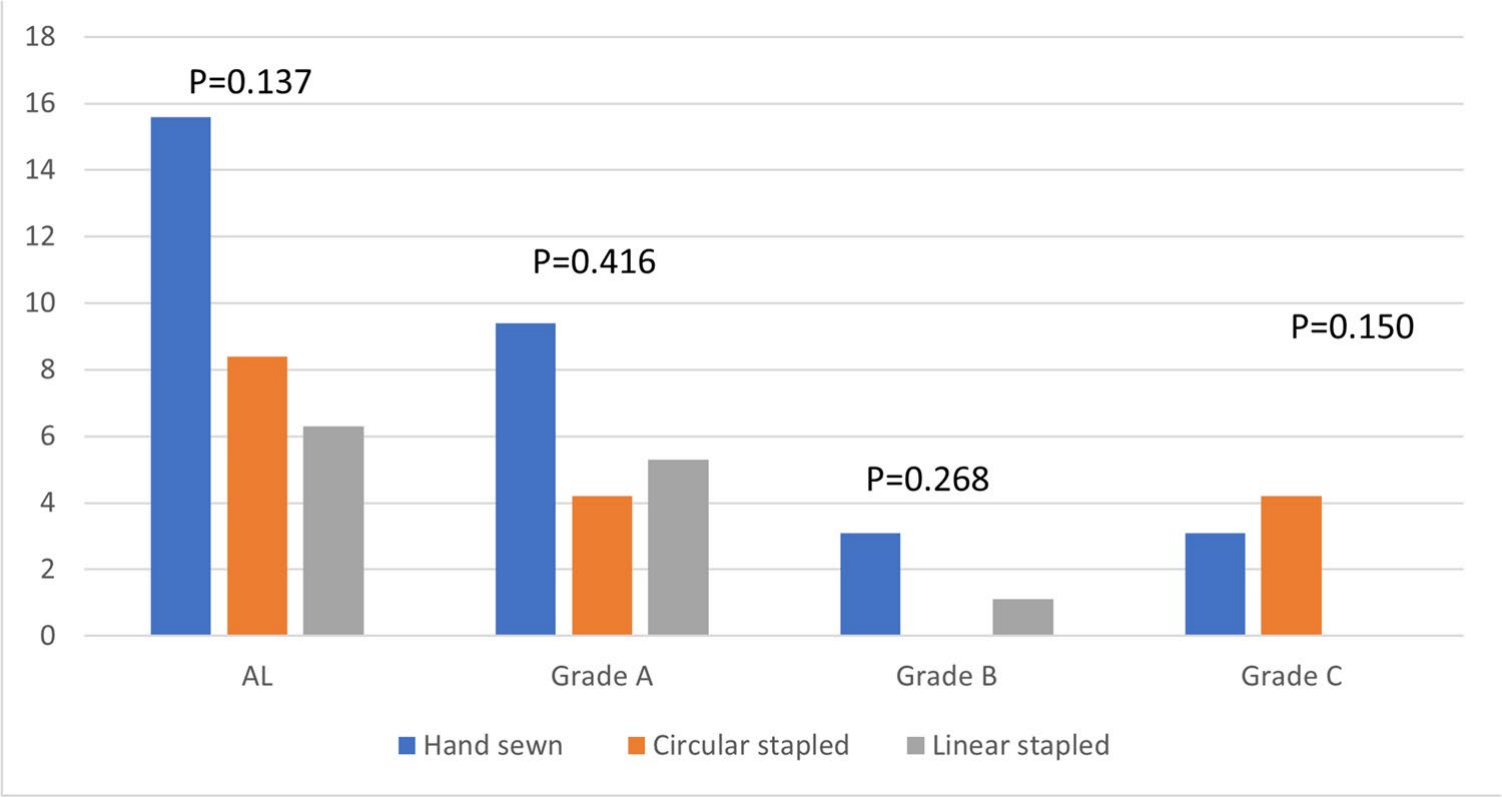
Rate of AL grade of the three compared group

Focusing on the first outcome, no significant differences were found between the three groups about = AL rate (*p* = 0.137), between the hand-sewn anastomosis group and the circular stapled anastomosis group (*p* = 0.198), between the circular-stapled anastomosis group and the linear-stapled anastomosis group (*p* = 0.599) and between the hand-sewn anastomosis group and the linear-stapled anastomosis group (*p* = 0.06). Moreover, considering the management of the AL (grade A: conservative treatment, grade B: invasive intervention other than repeat surgical intervention, grade C: surgical intervention) for each of the three groups, no significant differences were found. In details of the 14 leakages grade A, six belonged to the hand-sewn anastomosis group, three to the circular-stapled anastomosis group and five to the linear-stapled anastomosis group (*p* = 0.416), of the three leakages grade B, two belonged to the hand-sewn anastomosis group and one to the linear-stapled group (*p* = 0.268) and of the five leakages grade C, two belonged to the hand-sewn group and three to the circular-stapled group (*p* = 0.150) (Fig. 4).

Furthermore, the correlation between AL rate and the characteristics of anastomotic fashioning technique was evaluated. Particularly about the hand-sewn anastomotic technique, no significant differences were found between the two subgroups about the type of suture thread used (*p* = 0.110), regarding the circular-stapled anastomotic technique, no significant differences were found between the four subgroups about the circular-stapled technique (*p* = 0.763) and the stapler diameter (0.763) and about linear-stapled anastomotic technique, no significant differences were found between the nine subgroups about the stapler type (*p* = 0.093), the stapler length (*p* = 0.248), the layer of defect closing (*p* = 0.544) and the type of suture thread used for defect closing (*p* = 0.1) (Table 2).

**Table 2:** Correlation between the characteristics of each anastomotic technique and the rate of AL

	Suture characteristics	Leak	*p*-value
Hand-sewn anastomosis group	Barbed (*n* = 40) Non-braided (*n* = 24)	4 (10%) 6 (25%)	0.110
Circular stapled anastomosis group	Manual (*n* = 2) Purse string device (*n* = 69) Stapler diameter (25 mm) (*n* = 69) Stapler diameter (29 mm) (*n* = 2)	0 (0%) 3 (4.3%) 3 (4.3%) 0 (0%)	0.763 0.763
Linear stapled anastomosis group	Stapler type (mechanical) (*n* = 29) Stapler type (electric) (*n* = 66) Stapler length (45 mm) (*n* = 37) Stapler length (60 mm) (*n* = 5 8) Layer of defect closing (single) (*n* = 43) Layer of defect closing (double) (*n* = 52) Suture thread for defect closing (barbed) (*n* = 55) Suture thread for defect closing (braided) (*n* = 3) Suture thread for defect closing (non-braided) (*n* = 37)	0 (0%) 6 (9.1%) 1 (2.7%) 5 (8.6%) 2 (4.6%) 4 (7.7%) 4 (7.3%) 1 (33.3%) 1 (2.7%)	0.093 0.248 0.544 0.1

## Discussion

Radical esophagectomy with a complete lymphadenectomy is nowadays considered the standard treatment of the esophageal cancer [20].

Over the last decades, minimally invasive approaches have been gradually gaining favor among surgeons, given that it minimizes surgical trauma and optimizes postoperative outcomes with lower postoperative complication rate and similar oncologic results compared to conventional thoracotomy approaches [21–24].

Recently, robot-assisted minimally invasive esophagectomy (RAMIE) has been introduced as an alternative minimally invasive method which may allow improved the detection of thoracic structures and increased surgical precision [25]. In fact, the three-dimensional view and the EndoWrist® technology of robotic surgery have let to both a better visualization of the operative field and more accurate movements in narrow space [12–17]. Milone et al. has showed that robotic approach for the treatment of esophageal cancer could be considered superior to open approach, being guaranteed less postoperative complications and superior oncologic results, and that it could be considered slightly superior to laparoscopic surgery, providing less postoperative pneumonia and higher number of harvested nodes [18]. Recently, the International Upper Gastrointestinal International Robotic Association (UGIRA) has demonstrated that hybrid laparoscopic RAMIE and full RAMIE were oncologically equivalent with a potential decrease of postoperative complications and shorter (intensive care) stay after full RAMIE [26].

Nevertheless, anastomotic leakage AL remains a severe complication associated with esophagectomy [27]. Occurrence of an AL depends on several factors including nutritional, surgical and anesthesiological factors. Identification of risk factors for AL is of critical importance for prevention and treatment.

Surely, one of the most critical phases during esophagectomy is performing an intrathoracic esophagogastric anastomosis and no standardized anastomotic fashioning technique has yet been generally accepted. The approaches used involve hand-sewn-, circular- (CS) and linear-stapled (LS).

With limited robotic experience, the circular-stapled anastomotic technique might be the preferred approach [28] because of its relative reliability and simplicity [29], while the hand-sewn anastomotic technique or the linear-stapled anastomotic technique are more challenging due to their high technical requirements of suturing ability.

In order to clarify the knowledge about the results of different anastomotic techniques for esophagogastric anastomosis, we retrospectively compared outcomes of the hand-sewn-, linear- and circular-stapled techniques for patients undergoing TMIIL esophagectomy at seven high-volume Italian centers for upper gastrointestinal surgery.

The specific aim of this study is to evaluate the best intrathoracic esophagogastric anastomotic fashioning technique in the robotic setting.

Our results showed no significant differences about AL between the three groups. However, the linear-stapled group presented an incidence of AL (6.3%) lower than that showed by the hand-sewn anastomosis group (15.6%) and the circular-stapled anastomosis group (8.4%). It is interesting to highlight how the linear-stapled group with the highest number of included cases presented the lower incidence of AL (6.3%) according to Kingma BF et al. [30] who found a statistically significant reduction of the AL incidence in the linear-stapled group.

The linear-stapling technique typically results in a functional side-to-side (STS) anastomosis. Although evidences are not available, the SDS configuration could explain the lower incidence of AL due to some rilevant features such as the larger orifice size of the anastomosis and the direction of tension on the anastomosis. In detail, the larger orifice of the anastomosis facilitates intraluminal content passage and decreases circular pressure on the anastomosis. In addition, the weight of the gastric conduit directs the tension on the anastomosis sideways [30, 31].

These technical characterists could also explain the development in the linear-stapled anastomosis gruop of AL easier to trat conservatively. Our results showed indeed no leakages grade C in the linear-stapled anastomosis group (Fig. 4). It makes us to think that the better distribution of tension on the anastomosis could justify leakages which allow a conservative treatment due to their grade and to their less relevant clinical implications.

Moreover, the incidence of AL in the hand-sewn anastomosis group (15.6%) was the highest between the three compared groups.

Thus, our results suggested that the esophagogastric anastomosis performed using a linear stapler was associated to a decrease, although not significant, in AL compared with the levels occurring under other anastomotic techniques [32], and that the linear-stapled technique could be related to AL easier to treat conservatively.

However, being not reached a statistically significance in a multicenter experience, no standardized anastomotic fashioning technique has yet been generally accepted to perform the intrathoracic esophagogastric anastomosis. It remains one of the most technically difficult steps, and no way to perform the anastomosis is considered the best one; but nowadays, the robotic approach could help the surgeon given its 3D-magnified view with better ergonomics and lower physiologic tremor due to EndoWrist instruments.

## Conclusion

Our results seem to be in favour of the linear-stapled anastomosis technique but they should not be generalized due to study limitations. These limitations involve retrospective, non-randomized character of the study, the low sample size and the different intrathoracic esophagogastric anastomosis techniques chosen according to the surgeon’s preferences. Moreover, the available scientific evidence regarding both the comparison between the hand-sewn-, circular- (CS) and linear-stapling (LS) approaches and the anastomotic fashioning technique surgical outcomes is limited. Thus, it could be considered a call to perform ad hoc high-quality studies involving high-volume centers for upper gastrointestinal surgery to evaluate what is the most advantageous anastomotic technique and to give definitive conclusions.

## Data Availability

Data collected for the study are available on request from the corresponding author (Anna D’Amore) upon reasonable request.
